# Pre- to postoperative alpha-fetoprotein ratio-based nomogram to predict tumor recurrence in patients with hepatocellular carcinoma

**DOI:** 10.3389/fonc.2023.1134933

**Published:** 2023-04-14

**Authors:** Chengkai Yang, Huaxiang Wang, Jianyong Liu, Fang Yang, Lizhi Lv, Yi Jiang, Qiucheng Cai

**Affiliations:** ^1^ The Fuzong Clinical Medical College of Fujian Medical University, Fuzhou, China; ^2^ Department of Hepatobiliary and Pancreatic Surgery, Taihe Hospital, Affiliated Hospital of Hubei University of Medicine, Shiyan, China; ^3^ Department of Hepatobiliary Surgery, 900 Hospital of The Joint Logistics Team, Fuzhou, China

**Keywords:** alpha fetoprotein ratio, hepatocellular carcinoma, nomogram, recurrence free survival, curative resection

## Abstract

**Background:**

This study aimed to investigate the role of the alpha fetoprotein (AFP) ratio before and after curative resection in the prognosis of patients with hepatocellular carcinoma (HCC) and to develop a novel pre- to postoperative AFP ratio nomogram to predict recurrence free survival (RFS) for HCC patients after curative resection.

**Methods:**

A total of 485 pathologically confirmed HCC patients who underwent radical hepatectomy from January 2010 to December 2018 were retrospectively analyzed. The independent prognostic factors of hepatocellular carcinoma were identified by multivariate COX proportional model analysis, and the nomogram model was constructed. The receiver operating characteristic and the C-index were used to evaluate the accuracy and efficacy of the model prediction, the correction curve was used to assess the calibration of the prediction model, and decision curve analysis was used to evaluate the clinical application value of the nomogram model.

**Results:**

A total of 485 HCC patients were divided into the training cohort (n = 340) and the validation cohort (n = 145) by random sampling at a ratio of 7:3. Using X-tile software, it was found that the optimal cut-off value of the AFP ratio in the training cohort was 0.8. In both cohorts, the relapse-free survival of patients with an AFP ratio <0.8 (high-risk group) was significantly shorter than in those with an AFP ratio ≥0.8 (low-risk group) (P < 0.05). An AFP ratio <0.8 was an independent risk factor for recurrence of HCC after curative resection. Based on the AFP ratio, BCLC stage and cirrhosis diagnosis, a satisfactory nomogram was developed. The AUC of our nomogram for predicting 1-, 3-, and 5-year RFS was 0.719, 0.690, and 0.708 in the training cohort and 0.721, 0.682, and 0.681 in the validation cohort, respectively. Furthermore, our model demonstrated excellent stratification as well as clinical applicability.

**Conclusion:**

The AFP ratio was a reliable biomarker for tumor recurrence. This easy-to-use AFP ratio-based nomogram precisely predicted tumor recurrence in HCC patients after curative resection.

## 1 Introduction

Primary liver cancer is a common type of malignancy with rapid disease development, high aggressiveness, and poor prognosis, which seriously threatens human health and quality of life, of which hepatocellular carcinoma (HCC) accounts for more than 80% of cases (1). According to 2020 global cancer statistics, HCC is the sixth most common cancer and the third leading cause of cancer-related death in the world (2). For patients with early HCC, the radical treatment options include surgical resection, liver transplantation, and radiofrequency ablation. However, for most patients, radical resection, which is advantageous because of its high surgical resection rate and low mortality rate, is the preferred treatment. Unfortunately, the 5-year recurrence rate after radical resection of liver cancer is still high at 60%–70%, and the overall survival rate is still low (3–5).

To date, there have been many reports of the prognostic factors and prognostic models after curative resection of HCC patients, including the Barcelona Clinic Liver Cancer (BCLC) Staging System, American Joint Committee on Cancer (AJCC) Staging System, Cancer of the Liver Italian Program (CLIP) Staging System, Tokyo Scoring System, and Hong Kong Liver Cancer Staging System (6–10). In general, these predictive models have unique predictive performance; however, none are accepted (11–13).

Nobuoka et al. showed that changes in alpha-fetoprotein (AFP) levels before and after hepatectomy for HCC can effectively predict the postoperative prognosis (14). Another study also reported that pre- to postoperative changes in AFP levels are more reliable than changes in routine AFP levels as a prognostic indicator for monitoring recurrence after HCC hepatectomy (15). These studies indicate that the range of pre- to postoperative changes in AFP levels can be used as a predictive indicator of HCC.

Therefore, in this retrospective study, we confirmed the clinical significance of the pre- to postoperative AFP ratio in the postoperative prognosis of patients with HCC and developed a practical and novel nomogram for recurrence free survival (RFS) based on pre- to postoperative AFP ratios.

## 2 Methods

### 2.1 Study cohort and design

A total of 485 HCC patients who underwent hepatic resection at the 900th Hospital of Chinese People’s Liberation Army Joint Support Force from January 2010 to December 2018 were included. The inclusion criteria were as follows (1): confirmed diagnosis of primary HCC by two or more imaging modalities (ultrasound, computed tomography, or magnetic resonance imaging) or postoperative histopathologic examination (2); treated by intended cure resection, which was defined as negative margins with no residual tumor based on the histopathologic examination; and (3) well-documented clinical history and detailed follow-up information. The exclusion criteria were as follows (1): confirmed diagnosis of other malignant tumors (2); preoperative transarterial chemoembolization, radiofrequency ablation, or other anti-tumor therapy; and (3) perioperative death. A total of 1,655 patients were identified with primary hepatocellular carcinoma by the computerized medical record system. According to the inclusion and exclusion criteria, we identified 450 patients. They were stochastically dichotomized into the training cohort (n = 340) and the internal validation cohort (n = 145) at a ratio of 7:3. The study was approved by the ethics committee of the 900th Hospital of Chinese People’s Liberation Army Joint Support Force. All research procedures followed the relevant guidelines and regulations. Signed informed consent was obtained from all patients. This study was conducted in accordance with the Declaration of Helsinki.

### 2.2 Data acquisition

All data for this study were collected from each participant’s electronic medical record and included gender; age; white blood cell count (WBC); platelet count (PLT); levels of hemoglobin (HB), sodium (Na), alpha-fetoprotein (AFP), albumin (ALB), total bilirubin (Tbil), aspartate aminotransferase (AST), alkaline phosphatase (ALP), and alanine aminotransferase (ALT); tumor-node-metastasis (TNM) stage; Barcelona Clinic Liver Cancer (BCLC) stage; surgical information; and tumor characteristics.

### 2.3 Calculation of the alpha fetoprotein ratio

The AFP ratio was defined as the ratio of AFP values one week before hepatectomy to the lowest AFP values within 4 months after surgery. The normal range of AFP was defined as 0–20 ng/μL.

### 2.4 Follow-up

Patients received regular follow-up every 3–6 months. At each visit, detailed history and physical examination were performed, and AFP level, liver function, and images were re-examined, Abdominal ultrasound, computed tomography, or magnetic resonance imaging was performed. In some cases, the relevant examinations were performed earlier than scheduled to identify patients with suspected intrahepatic and extrahepatic recurrence and metastasis. Postoperative patients received standardized treatment and regular follow-up, and treatment plans were adjusted if necessary (16). The end point of the follow-up was tumor recurrence, death, or last follow up.

### 2.5 Statistical analysis

Recurrence free survival was measured from the date of surgery to the date of recurrence, the date of death, or the study closure date of December 31, 2021. Basic descriptive statistics, including mean, median, standard deviation (SD), and frequency (percentage), were used to characterize the dataset in both training and validation cohorts. Measurement data were presented as median or mean ± SD. Mann-Whitney U test was used to compare continuous variables and chi-square or Fisher’s exact test was used to compare categorical variables. X-tile statistical software (17) (version 3.6.1, Yale University, New Haven, CT, USA) was applied to determine the optimal threshold of the AFP ratio for RFS. According to the defined cutoff value, patients were divided into the low AFP ratio group or the high AFP ratio group, and the Kaplan-Meier method was used to draw survival curves. The training cohort was used to generate the nomogram based on multivariate regression to predict the 1-, 3-, and 5-year RFS using the “rms” package. The Web calculator was built by the “shiny” package. The area under the receiver operating characteristic curve and the C-index were used to evaluate the accuracy of the model in predicting survival, and the larger the C-index, the higher the accuracy of the model. Each patient had a total risk score (NomoScore: nomogram risk score) for risk stratification of RFS according to the nomogram. Patients were divided into different risk groups (low-, moderate-, high-) with the cut-off points automatically calculated using X-tile software. Decision curve analysis (DCA) was conducted to determine the clinical benefit of the nomogram by quantifying the net benefits along with the increase in threshold probabilities. Kaplan-Meier curves were plotted using GraphPad Prism 8.0 software. P < 0.05 was considered statistically significant.

## 3 Results

**Figure 1:**
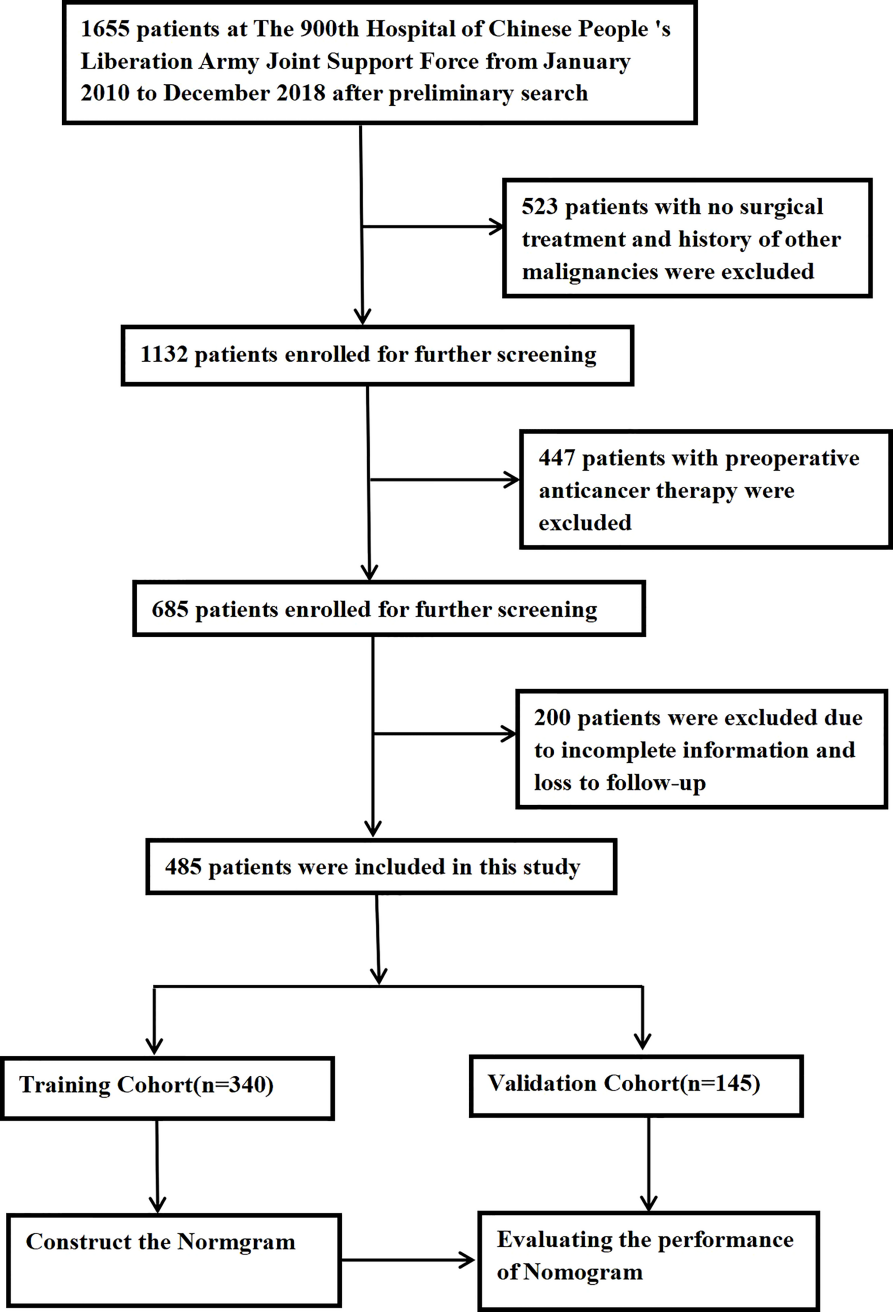
Flow chart of the process of patient selection.

A total of 1,655 patient records in the hospital medical record system were reviewed. According to the exclusion criteria, 485 patients were included in our study cohort and randomized at a ratio of 7:3 into the training group (n = 340) and the validation group (n = 145). The process of patient selection is illustrated in **Figure 1**.

A total of 485 patients diagnosed with HCC were included, with a median follow-up time of 137 months (range, 1–275 months). There was no significant difference in any clinical parameters between the training and validation groups (**Table 1**).

**Table 1:** Clinical characteristics of the training group and the validation group.

Variables	Training cohort (N=340)	Validation cohort (N=145)	X^2^	P
Sex			3.508	0.061
Male	288(84.7%)	132(91.0%)		
Female	52(15.3%)	13(9.0%)		
Age, years			1.390	0.238
≥60	90(26.5%)	46(31.7%)		
<50	250(73.5%)	99(68.3%)		
WBC, ×10^9^/L			0.729	0.393
>10	25(7.4%)	14(9.7%)		
≤10	315(92.6%)	131(90.3%)		
HB, g/L			1.208	0.272
≥120	311(91.5%)	128(88.3%)		
<120	29(8.5%)	17(11.7%)		
PLT, ×10^9^/L			0.758	0.384
≥100	311(91.5%)	129(89.0%)		
<100	29(8.5%)	16(11.0%)		
Na, mmol/L			1.333	0.248
≥135	327(96.2%)	136(93.8%)		
<135	13(3.8%)	9(6.2%)		
ALT, U/L			0.596	0.440
>50	96(28.2%)	36(24.8%)		
≤50	244(71.8%)	109(75.2%)		
AST, U/L			0.577	0.448
>40	132(38.8%)	51(35.2%)		
≤40	208(61.2%)	94(64.8%)		
Tbil, U/L			0.319	0.572
>17.1	90(26.5%)	42(29.0%)		
≤17.1	250(73.5%)	103(71.0%)		
ALB, g/L			0.246	0.620
≥30	337(99.1%)	143(98.6%)		
<30	3(0.9%)	2(1.4%)		
γ-GT, U/L			0.814	0.367
≥50	177(52.1%)	69(47.6%)		
<50	163(47.9%)	76(52.4%)		
HBsAg			0.059	0.808
Positive	305(89.7%)	129(89.0%)		
Negative	35(10.3%)	16(11.0%)		
HBV-DNA			2.368	0.124
>500	212(62.4%)	101(69.7%)		
≤500	128(37.6%)	44(30.3%)		
Cirrhosis			0.044	0.833
Positive	163(47.9%)	68(46.9%)		
Negative	177(52.1%)	77(53.1%)		
Number of tumors			0.793	0.373
Solitary	277(81.5%)	123(84.8%)		
Multiple	63(18.5%)	22(15.2%)		
Tumor size, cm			0.341	0.559
≥5	145(42.6%)	66(45.5%)		
<5	195(57.4%)	79(54.5%)		
MVI			0.373	0.541
Positive	78(22.9%)	37(25.5%)		
Negative	262(77.1%)	108(74.5%)		
AJCC T stage			0.527	0.468
I	138(40.6%)	64(44.1%)		
II, III	202(59.4%)	81(55.9%)		
BCLC stage			0.074	0.786
0, A	241(70.9%)	101(69.7%)		
B, C	99(29.1%)	44(30.3%)		
Pathological differentiation			0.070	0.791
Poorly	28(8.2%)	13(9.0%)		
Moderately and well	312(91.8%)	132(91.0%)		
AFP ratio			0.830	0.362
≥8	303(89.1%)	125(86.2%)		
<8	37(10.9%)	20(13.8%)		

WBC, white blood cells; HB, hemoglobin; PLT, platelets; Na, Na ion; ALT, alanine aminotransferase; AST, aspartate transaminase; Tbil, total bilirubin; ALP, alkaline phosphatase; HBsAg, hepatitis B surface antigen; HBV, hepatitis B virus; MVI, microvascular invasion; AFP ratio, alpha-fetoprotein ratio; AJCC, American Joint Committee on Cancer; BCLC, Barcelona Clinic Liver Cancer.

### 3.1 Relationship between the alpha fetoprotein ratio and clinical characteristics in patients with hepatocellular carcinoma

Using X-tile software, the optimal cutoff value of the AFP ratio for the training group was 0.8, which was divided into high-risk (AFP ratio <0.8) and low-risk (AFP ratio ≥0.8) groups using 0.8 as the cut-off threshold. The correlation between the AFP ratio and other characteristics is shown in **Table 2**. In the training group, there were significant differences in HBsAg positivity, MVI positivity, and BCLC stage between the high AFP ratio and low AFP ratio groups (P = 0.016, 0.007, 0.017, respectively). In the validation group, there was a significant difference in BCLC stage between the high AFP ratio and low AFP ratio groups (P = 0.031). As predicted, the AFP levels before hepatectomy of the high AFP ratio group were higher than those of the low AFP ratio group. In both cohorts, there were no apparent differences in other clinical and laboratory parameters between the two groups with high and low AFP ratios.

**Table 2:** Clinical features of patients with high and low AFP ratios in two cohorts.

Variables	Training cohort	AFP ratio low (n=37)	p value	Validation cohort	AFP ratio low (n=20)	p value
	AFP ratio high (n=303)			AFP ratio high (n=125)		
Sex			0.422			0.504
Male	255(84.2%)	33(89.2%)		113(90.4%)	19(95.0%)	
Female	48(15.8%)	4(10.8%)		12(9.6%)	1(5.0%)	
Age, years			0.206			0.392
≥60	77(25.4%)	13(35.1%)		38(30.4%)	8(40.0%)	
<60	226(74.6%)	24(64.9%)		87(69.6%)	12(60.0%)	
WBC, ×10^9^/L			0.852			0.448
>10	22(7.3%)	3(8.1%)		13(10.4%)	1(5.0%)	
≤10	281(92.7%)	34(91.9%)		112(89.6%)	19(95.0%)	
HB, g/L			0.599			0.215
≥120	278(91.7%)	33(89.2%)		112(89.6%)	16(80.0%)	
<120	25(8.3%)	4(10.8%)		13(10.4%)	4(20.0%)	
PLT, ×10^9^/L			0.179			0.168
≥100	275(90.8%)	36(97.3%)		113(90.4%)	16(80.0%)	
<100	28(9.2%)	1(2.7%)		12(9.6%)	4(20.0%)	
Na, mmol/L			0.595			0.810
≥135	292(96.4%)	35(94.6%)		117(93.6%)	19(95.0%)	
<135	11(3.6%)	2(5.4%)		8(6.4%)	1(5.0%)	
ALT, U/L			0.863			0.985
>50	86(28.4%)	10(27.0%)		31(24.8%)	5(25.0%)	
≤50	217(71.6%)	27(73.0%)		94(75.2%)	15(75.0%)	
AST, U/L			0.346			0.602
>40	115(38.0%)	17(45.9%)		45(36.0%)	6(30.0%)	
≤40	188(62.0%)	20(54.1%)		80(64.0%)	14(70.0%)	
Tbil, U/L			0.754			0.341
>17.1	81(26.7%)	9(24.3%)		38(30.4%)	4(20.0%)	
≤17.1	222(73.3%)	28(75.7%)		87(69.6%)	16(80.0%)	
ALB, g/L			0.543			0.135
≥30	300(99.0%)	37(100.0%)		124(99.2%)	19(95.0%)	
<30	3(0.9%)	0(0.0%)		1(0.8%)	1(5.0%)	
γ-GT, U/L			0.340			0.475
≥50	155(51.2%)	22(59.5%)		58(46.4%)	11(55.0%)	
<50	148(48.8%)	15(40.5%)		67(53.6%)	9(45.0%)	
HBsAg			0.016			0.542
Positive	276(91.1%)	29(78.4%)		112(89.6%)	17(85.0%)	
Negative	27(8.9%)	8(21.6%)		13(10.4%)	3(15.0%)	
HBV-DNA			0.068			0.971
>500	194(64.0%)	18(48.6%)		87(69.6%)	14(70.0%)	
≤500	109(36.0%)	19(51.3%)		38(30.4%)	6(30.0%)	
Cirrhosis			0.660			0.855
Positive	144(47.5%)	19(51.4%)		59(47.2%)	9(45.0%)	
Negative	159(52.5%)	18(48.6%)		66(52.8%)	11(55.0%)	
Number of tumors			0.406			0.517
Solitary	245(80.9%)	32(86.5%)		107(85.6%)	16(80.0%)	
Multiple	58(19.1%)	5(13.5%)		18(14.4%)	4(20.0%)	
Tumor size, cm			0.434			0.161
≥5	127(41.9%)	18(48.6%)		54(43.2%)	12(60.0%)	
<5	176(58.1%)	19(51.4%)		71(56.8%)	8(40.0%)	
MVI			0.007			0.542
Positive	63(20.8%)	15(40.5%)		33(26.4%)	4(20.0%)	
Negative	240(79.2%)	22(59.5%)		92(73.6%)	16(80.0%)	
AJCC T stage			0.474			0.375
I	125(41.3%)	13(35.1%)		57(45.6%)	7(35.0%)	
II, III	178(58.7%)	24(64.9%)		68(54.4%)	13(65.0%)	
BCLC stage			0.017			0.031
0, A	221(72.9%)	20(54.1%)		87(69.6%)	9(45.0%)	
B, C	82(27.1%)	17(45.9%)		38(30.4%)	11(55.0%)	
Pathological differentiation			0.216			0.063
Poorly	23(7.6%)	5(13.5%)		9(7.2%)	4(20.0%)	
Moderately and well	280(92.4%)	32(86.5%)		116(92.8%)	16(80.0%)	
AFP before hepatectomy (ng/μl)	79.50(7.40-1036.00)	2.20(1.08-13.22)	0.000	149.52(8.66-1247.00)	5.35(2.48-213.68)	0.002
AFP after hepatectomy (ng/μl)	5.30 (2.78-12.80)	6.00(2.98-36.90)	0.565	6.05(2.62-213.68)	8.65(2.68-620.67)	0.238

WBC, white blood cells; HB, hemoglobin; PLT, platelets; Na, Na ion; ALT, alanine aminotransferase; AST, aspartate transaminase; Tbil, total bilirubin; ALP, alkaline phosphatase; HBsAg, hepatitis B surface antigen; HBV, hepatitis B virus; MVI, microvascular invasion; AFP alpha-fetoprotein; AFP ratio, alpha-fetoprotein ratio; AJCC, American Joint Committee on Cancer; BCLC, Barcelona Clinic Liver Cancer.

### 3.2 Prognostic value of the alpha fetoprotein ratio in patients with hepatocellular carcinoma

**Figure 2:**
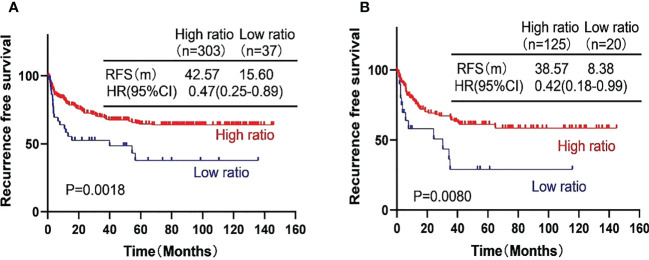
Kaplan-Meier curves showing the recurrence free survival (RFS) of the high-risk subgroup and the low-risk subgroup of hepatocellular carcinoma patients in the training cohort **(A)** and the validation cohort **(B)**.

We further investigated the prognostic value of the AFP ratio in HCC patients after curative resection. In the training cohort, the RFS was 42.57 months with high AFP ratio and 15.60 months with low AFP ratio (p = 0.0018; HR, 0. 47, 95% CI, 0.25–0.88). In the validation cohort, the RFS was 38.57 months with high AFP ratio and 8.38 months with low AFP ratio (p = 0.0080; HR, 0.42, 95% CI, 0.18–0.99) (**Figure 2**).

### 3.3 Univariate and multivariate analyses

The Cox proportional hazards regression model was used to analyze the AFP ratio and clinical parameters in patients with HCC, and variables with P < 0.05 were included in the multivariate Cox regression analysis. In the training cohort, the results of univariate analysis showed that AFP ratio, tumor capsule, MVI, BCLC stage, and cirrhosis diagnosis were significantly associated with RFS, whereas the results of multivariate analysis showed that AFP ratio, BCLC stage, and cirrhosis diagnosis were important independent factors affecting prognosis. In the validation cohort, univariate and multivariate analyses confirmed that AFP ratio was an independent risk factor for patients with HCC. These results are presented in **Tables 3**, **4**.

**Table 3:** Univariate and multivariate analyses for recurrence free survival in the training cohort.

Variables	Univariate Analysis		Multivariate Analysis	
	HR (95% CI)	P	HR (95% CI)	P
Sex	1.146 (0.758–1.735)	0.518		
Age, years	0.855 (0.609–1.199)	0.363		
WBC, ×10^9^/L	1.117 (0.659–1.893)	0.681		
HB, g/L	1.384 (0.861–2.227)	0.180		
PLT, ×10^9^/L	1.251 (0.769–2.034)	0.367		
Na, mmol/L	0.915 (0.406–2.064)	0.830		
ALT, U/L	1.268 (0.932–1.727)	0.131		
AST, U/L	1.793 (1.344–2.392)	0.000	1.271 (0.923–1.749)	0.141
Tbil, U/L	1.134 (0.824–1.562)	0.440		
ALB, g/L	0.049 (0.000–15.254)	0.303		
γ-GT, U/L	1.770 (1.321–2.372)	0.000	1.288 (0.932–1.779)	0.125
HBsAg	0.981 (0.610–1.576)	0.935		
HBV-DNA	1.091 (0.807–1.473)	0.572		
Cirrhosis	1.430 (1.074–1.904)	0.014	1.648 (1.217–2.231)	0.001
Number of tumors	1.733 (1.235–2.431)	0.001	0.814 (0.471–1.407)	0.461
Tumor size, cm	1.721 (1.291–2.294)	0.000	1.032 (0.638–1.672)	0.897
MVI	2.145 (1.563–2.945)	0.000	0.476 (0.191–1.184)	0.110
AJCC T stage	1.912 (1.514–2.415)	0.000	1.317 (0.771–2.252)	0.314
BCLC stage	1.550 (1.350–1.779)	0.000	1.929 (1.211–3.073)	0.006
Pathological differentiation	0.918 (0.541–1.557)	0.751		
AFP ratio	2.074 (1.402–3.068)	0.000	0.533(0.358–0.791)	0.002

WBC, white blood cells; HB, hemoglobin; PLT, platelets; Na, Na ion; ALT, alanine aminotransferase; AST, aspartate transaminase; Tbil, total bilirubin; ALP, alkaline phosphatase; HBsAg, hepatitis B surface antigen; HBV, hepatitis B virus; MVI, microvascular invasion; AFP ratio, alpha-fetoprotein ratio; AJCC, American Joint Committee on Cancer; BCLC, Barcelona Clinic Liver Cancer.

**Table 4:** Univariate and multivariate analyses for recurrence free survival in the validation cohort.

Variables	Univariate Analysis		Multivariate Analysis	
	HR (95% CI)	P	HR (95% CI)	P
Sex	1.741 (0.760–3.987)	0.190		
Age, years	0.976 (0.625–1.524)	0.917		
WBC, ×10^9^/L	1.060 (0.549–2.046)	0.862		
HB, g/L	1.391 (0.756–2.559)	0.289		
PLT, ×10^9^/L	0.993 (0.514–1.918)	0.983		
Na, mmol/L	1.048 (0.457–2.400)	0.912		
ALT, U/L	1.157 (0.720–1.858)	0.547		
AST, U/L	1.589 (1.042–2.421)	0.031	1.601 (1.042–2.462)	0.032
Tbil, U/L	1.159 (0.745–1.803)	0.514		
ALB, g/L	1.826 (0.447–7.466)	0.402		
γ-GT, U/L	1.417 (0.938–2.139)	0.097		
HBsAg	1.527 (0.738–3.159)	0.253		
HBV-DNA	1.154 (0.732–1.819)	0.538		
Cirrhosis	1.267 (0.984–1.631)	0.067		
Number of tumors	1.265(0.727–2.203)	0.405		
Tumor size, cm	1.926 (1.270–2.919)	0.002	1.457 (0.731–2.905)	0.285
MVI	2.014 (1.299–3.122)	0.002	0.364(0.111–1.201)	0.097
AJCC T stage	1.636 (1.176–2.274)	0.003	0.695 (0.361–1.339)	0.277
BCLC stage	1.436 (1.194–1.727)	0.000	2.248 (1.239–4.081)	0.008
Pathological differentiation	1.137 (0.571–2.264)	0.714		
AFP ratio	2.027 (1.159–3.547)	0.013	2.248(1.361–4.406)	0.003

WBC, white blood cells; HB, hemoglobin; PLT, platelets; Na, Na ion; ALT, alanine aminotransferase; AST, aspartate transaminase; Tbil, total bilirubin; ALP, alkaline phosphatase; HBsAg, hepatitis B surface antigen; HBV, hepatitis B virus; MVI, microvascular invasion; AFP ratio, alpha-fetoprotein ratio; AJCC, American Joint Committee on Cancer; BCLC, Barcelona Clinic Liver Cancer.

### 3.4 Construction and validation of alpha fetoprotein ratio-based nomogram for recurrence free survival

**Figure 3:**
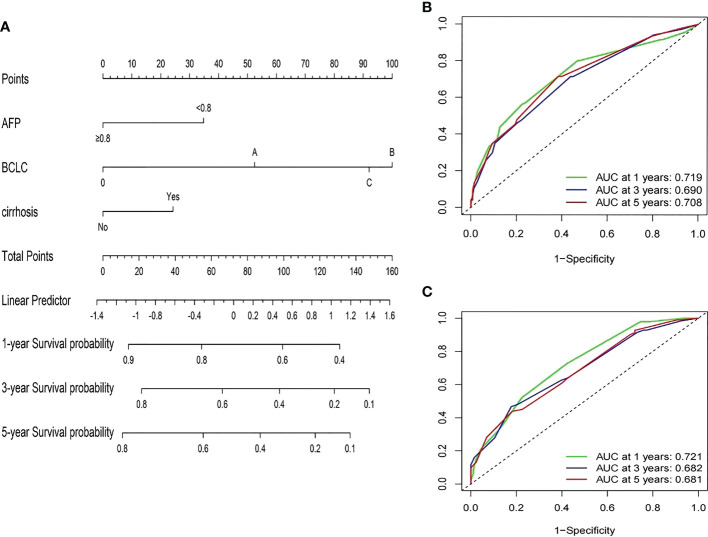
Developed prognosis nomogram model for RFS. **(A)** nomogram for predicting 1-, 3-, and 5-year RFS of HCC patients after curative resection. **(B, C)** 1-, 3-, and 5-year ROC values of RFS in the training cohort **(B)** and the validation cohort **(C)**.

Based on the multivariate Cox proportional hazards model of the training group, three variables, including AFP ratio, BCLC stage, and cirrhosis diagnosis, were used to establish satisfactory nomograms for predicting 1-, 3-, and 5-year RFS in HCC patients (**Figure 3A**). The AUC values for our nomogram for predicting 1-, 3-, and 5-year RFS were 0.719, 0.690, and 0.708 in the training cohort and 0.721, 0.682, and 0.681 in the validation cohort, respectively (**Figures 3B, C**).

### 3.5 C-index and calibration plot in the training and validation cohorts

**Figure 4:**
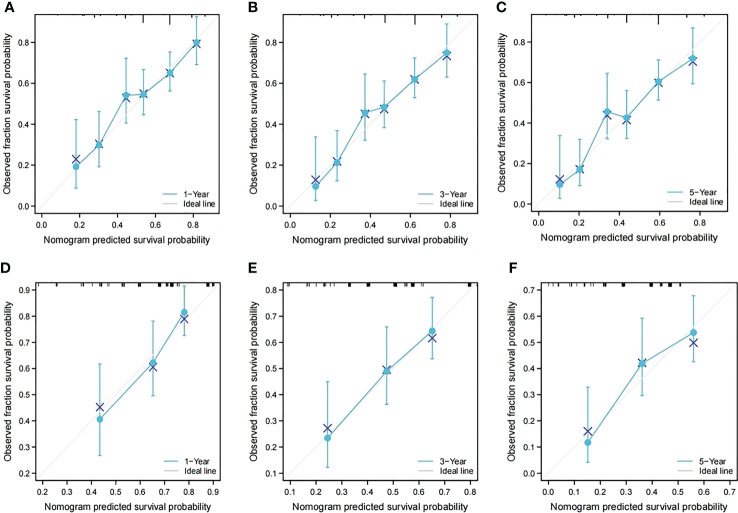
Calibration curves of the prognostic nomogram in both groups. **(A)** Calibration curve of the nomogram for the training cohort at 1 year. **(B)** Calibration curve of the nomogram for the training cohort at 3 years. **(C)** Calibration curve of the nomogram for the training cohort at 5 years. **(D)** Calibration curve of the nomogram for the validation cohort at 1 year. **(E)** Calibration curve of the nomogram for the validation cohort at 3 years. **(F)** Calibration curve of the nomogram for the validation cohort at 5 years.

In the training cohort, the C-index of the nomogram for the RFS prediction was 0.67, and the calibration curves for the 1-, 3- and 5-year RFS rates overlapped with the standard lines, suggesting excellent agreement between predicted and actual RFS values (**Figures 4A–C**). In the validation cohort, the C-index of the nomogram for the RFS prediction was 0.64, and the calibration curves for the 1-, 3- and 5-year RFS rates overlapped with the standard lines, also suggesting excellent agreement between predicted and actual RFS values (**Figures 4D–F**).

### 3.6 Decision curve analysis for clinical utility of the nomogram

**Figure 5:**
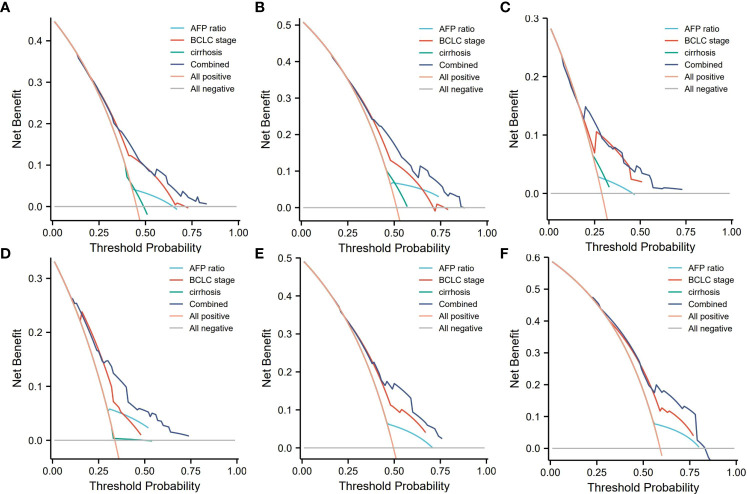
Decision curve analysis (DCA) of the nomogram. **(A–C)** DCA of 1-, 3-, and 5-year RFS predicted nomograms in the training cohort; **(D–F)** DCA of the 1-, 3-, and 5-year RFS predicted nomograms in the validation cohort. AFP ratio, alpha-fetoprotein ratio; BCLC, the Barcelona Clinic Liver Cancer staging system.

Decision curve analysis can determine the clinical benefit of a nomogram by quantifying the net benefit as well as the increase in threshold probability. The DCA of our nomogram and the independent risk factors, namely, AFP ratio, BCLC stage, and cirrhosis diagnosis, in the training and validation cohorts for 1-, 3-, and 5-year RFS are illustrated in **Figure 5**. Both the training and validation groups showed that, compared with AFP ratio, BCLC stage, cirrhosis diagnosis, our nomogram provided a better clinical benefit and had significant clinical application in the prediction of HCC recurrence (**Figures 5A–F**).

### 3.7 Prognostic assessment and risk stratification

**Figure 6:**
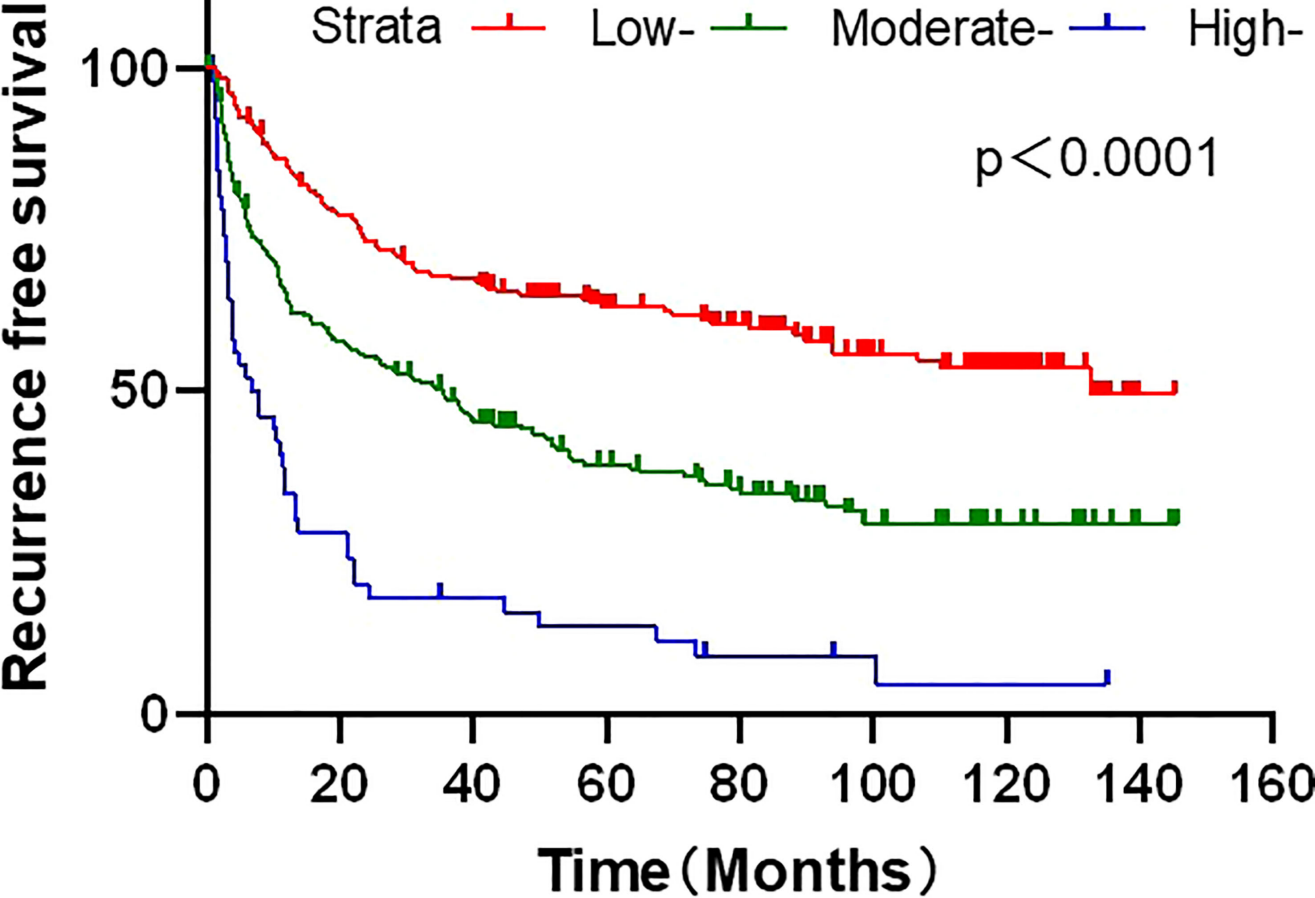
Risk stratification of the developed nomogram model for recurrence free survival.

We further examined nomograms for prognostic evaluation and risk stratification. Each HCC patient received a different score according to the total risk score calculated from the nomogram model, which was divided into different risk groups to determine the discriminative ability of the nomogram for RFS. The optimal cut-off points were automatically calculated by X-tile software. The calculated risk score could classify HCC patients into high-risk (>115), middle-risk (35–114), and low-risk (<34) groups. The Kaplan-Meier method was used to compare the RFS of three different risk groups, and the results showed that the nomogram risk score had a significant discriminatory ability for the risk of patient recurrence (P < 0.05, **Figure 6**).

## 4 Discussion

Hepatocellular carcinoma is a common type of malignancy, and its methods of treatment and effects of treatment are not the same, of which curative resection surgery is the generally accepted treatment of choice (18). In-depth studies have focused on the development, progression, and prognostic factors of HCC, and several improvements have been reported in diagnosis and treatment, surgical techniques, and comprehensive treatment; however, the prognosis of HCC after treatment is still not satisfactory. Studies have demonstrated that the 5-year recurrence and metastasis rate after surgical resection of HCC can be as high as 70% (19), and the 5-year overall survival rate is only 39%–65% (20, 21). The high recurrence rate of HCC after surgical resection is unacceptable, because it affects the prognosis of millions of HCC patients worldwide, so the development of a scoring model that can accurately predict the postoperative recurrence of HCC patients can help clinicians to individualize the preoperative treatment methods, surgical methods, and postoperative treatment options, which is critical for improving the long-term survival of HCC patients.

Alpha-fetoprotein is a serum biomarker used in the clinical diagnosis and postoperative monitoring of HCC, and the positive detection rate in HCC patients is approximately 70%–80%. In HCC patients with a negative detection rate, AFP is still an important indicator for predicting the prognosis of HCC (22, 23). Previous studies have demonstrated that high AFP levels in HCC patients are significantly associated with poor prognosis (24, 25). Some liver cancer staging systems, such as the Biomarker Combined Japan Integrated Staging (bm-JIS) and the CLIP score, also utilize serum AFP levels (8, 26), whereas AFP levels ≤400 ng/mL are included in the Hangzhou criteria as a patient selection criterion before liver transplantation (27).

However, the effectiveness of preoperative or postoperative AFP levels alone in predicting recurrence after HCC resection remains inadequate. Serum AFP levels may reflect tumor burden, and a decrease in the serum AFP level after surgery is considered to indicate a good response to treatment (28, 29). Thus, dynamic changes between preoperative and postoperative AFP levels may predict HCC prognosis more accurately than preoperative or postoperative AFP levels alone. Toro et al. indicated that AFP levels before and after treatment were associated with survival in HCC patients (30), whereas Nobuoka et al. showed that AFP levels changed from positive preoperatively to negative postoperatively and could be used to predict postoperative recurrence of HCC (14). Luo et al. also demonstrated that changes in preoperative to postoperative AFP levels could be used to assess recurrence and survival after radiofrequency ablation in HCC patients, and that the preoperative to postoperative AFP ratio could be used as a potential assessment index (31). Taken collectively, these findings suggested a strong association between changes in AFP levels before and after treatment and HCC prognosis.

In this study, we confirmed the prognostic value of the ratio of preoperative AFP to postoperative AFP in HCC patients after surgery. The optimal cut-off value of the AFP ratio in this study was 0.83, and HCC patients with AFP ratios less than 0.8 had significantly lower recurrence-free survival than those with AFP ratios greater than 0.8 in training and validation groups. Univariate and multivariate analyses showed that AFP ratio was an independent risk predictor for postoperative RFS in HCC patients. In conclusion, our results indicated that the AFP ratio was an important prognostic indicator for HCC patients undergoing curative resection surgery.

Nomograms transform complex regression equations into visual graphs, and they are reliable tools for integrating and quantifying significant risk factors for the prognosis of a variety of diseases (32, 33). Accurate prognostic evaluations can help physicians follow patients and select individualized treatment measures based on risk-benefit assessment scores. Our study developed a novel nomogram based on independent risk predictors of postoperative RFS in HCC patients. The nomogram included three variables, namely, AFP ratio, BCLC stage, and cirrhosis. Currently, BCLC staging is the most widely used staging system worldwide, and its unique advantage lies in the comprehensive consideration of the general condition, tumor condition, and liver function of HCC patients, and the preferred treatment is proposed according to the stage (34). Several studies have reported that the BCLC stage was an independent factor for the prognosis of HCC patients, which has better survival stratification and prognostic ability than other liver cancer staging modalities such as TNM stage, Japan Integrated Staging (JIS), and CLIP score (35, 36). Cirrhosis, a chronic persistent liver injury, is the result of multiple etiologies that lead to hepatocyte necrosis, which in turn causes severe liver lesions, thus becoming a risk factor for early HCC and postoperative recurrence. Many prognostic studies have demonstrated that cirrhosis was an independent predictor of HCC prognosis (37, 38). Our study confirmed BCLC stage and cirrhosis as independent and significant risk factors for poor disease-free survival after curative liver resection.

In this study, we combined the prediction model constructed by the pre- to postoperative AFP ratio, BCLC stage, and cirrhosis diagnosis, and comprehensively considered the comprehensive effects of three major aspects, namely, laboratory examination indicators, clinical characteristics, and imaging characteristics. The C-index of the model in the training group was 0.67, and the AUC values for predicting 1-, 3-, and 5-year RFS were 0.719, 0.690, and 0.708, respectively. In the validation group, the C-index was 0.64, and the AUC values for predicting 1-, 3-, and 5-year RFS were 0.721, 0.682, and 0.681, respectively. In addition, DCA showed that our nomogram achieved a higher net benefit than any single prognostic indicator such as BCLC stage and cirrhosis diagnosis in predicting 1-, 3-, and 5-year RFS. In summary, the nomogram prediction model we created based on the AFP ratio had high reliability and high clinical application value, and it can help clinicians individualize the treatment of HCC patients.

Although our nomogram showed satisfactory clinical performance, many limitations remain. First, this investigation was a single-center study, and the sample size was insufficient, which may bias the study results. Second, retrospective study analysis associated with selection bias in data collection and postoperative follow-up, and finally, in this study, the main cause of HCC was hepatitis B virus infection, which is different from the common cause in Western countries, which may affect AFP secretion. Thus, in the future, this model requires larger cohorts and prospective studies to validate its stratification strategy and prognostic ability.

## 5 Conclusion

In conclusion, the AFP ratio-based RFS nomogram prognostic model showed great potential predictive accuracy in HCC patients after curative resection and could be used in clinical practice to accurately assess RFS and identify high-risk patients, so as to develop more precise individualized treatment plans, and then increase the survival time of patients.

## Data availability statement

The original contributions presented in the study are included in the article/**Supplementary Material**. Further inquiries can be directed to the corresponding authors.

## Ethics statement

Written informed consent was obtained from the individual(s) for the publication of any potentially identifiable images or data included in this article.

## Author contributions

CY and HW collected and statistically analyzed the medical records, as well as wrote the manuscript. JL and FY collected medical records and performed data analysis. LL, YJ, and QC provided practical advice, grant support, and administrative support, and critically revised the manuscript. All authors contributed to the article and approved the submitted version.
